# 5-(Diphenyl­methyl­idene)pyrrolidin-2-one

**DOI:** 10.1107/S1600536812043851

**Published:** 2012-10-27

**Authors:** Tzu-Fang Hsu, Yan-Ru Chen, Bor-Hunn Huang, Ming-Jen Chen

**Affiliations:** a Department of Applied Cosmetology and Graduate Institute of Cosmetic Science, Hungkuang University, Taichung 433, Taiwan; b Department of Chemistry, National Chung Hsing University, Taichung 402, Taiwan

## Abstract

In the title compound, C_17_H_15_NO, the dihedral angle between the phenyl rings is 80.1 (2)°. In the crystal, mol­ecules are linked by pairs of N—H⋯O hydrogen bonds, forming inversion dimers.

## Related literature

The title compound is a pyrrolidin-2-one derivative. For the preparation of related structures, see: Fujihara & Tomioka (1999 ▶); Enders & Han (2008 ▶). For a related structure containing inter­molecular N—H⋯O=C hydrogen bonds, see: Asiri *et al.* (2012 ▶).
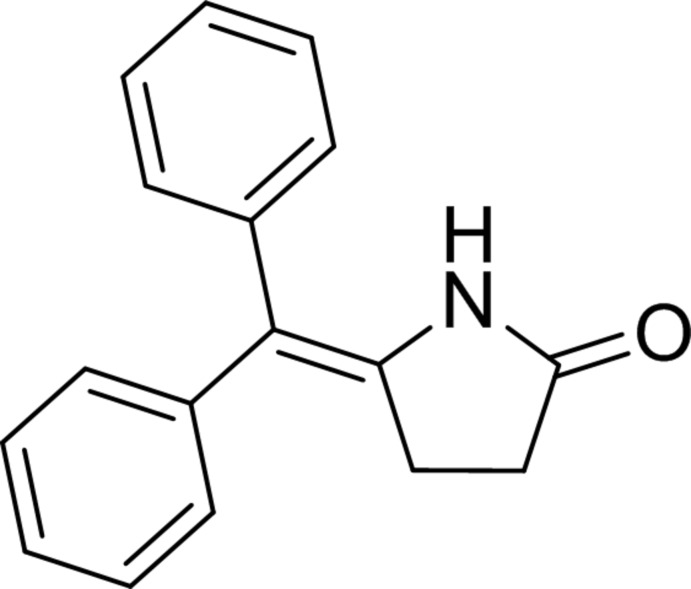


## Experimental

### 

#### Crystal data

C_17_H_15_NO
*M* *_r_* = 249.30
Triclinic, 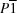

*a* = 7.135 (2) Å
*b* = 7.885 (2) Å
*c* = 12.184 (4) Å
α = 89.76 (3)°
β = 75.09 (3)°
γ = 85.65 (2)°
*V* = 660.4 (4) Å^3^
*Z* = 2
Mo *K*α radiation
μ = 0.08 mm^−1^
*T* = 293 K
0.70 × 0.50 × 0.35 mm

#### Data collection

Agilent Xcalibur (Sapphire3, Gemini) diffractometer
Absorption correction: multi-scan (*CrysAlis PRO*; Agilent, 2011 ▶) *T*_min_ = 0.542, *T*_max_ = 1.000
5549 measured reflections
2999 independent reflections
1951 reflections with *I* > 2σ(*I*)
*R*_int_ = 0.052

#### Refinement

*R*[*F* ^2^ > 2σ(*F* ^2^)] = 0.074
*wR*(*F* ^2^) = 0.225
*S* = 1.06
2999 reflections
172 parameters
H-atom parameters constrained
Δρ_max_ = 0.31 e Å^−3^
Δρ_min_ = −0.26 e Å^−3^

### 

Data collection: *CrysAlis PRO* (Agilent, 2011 ▶); cell refinement: *CrysAlis PRO*; data reduction: *CrysAlis PRO*; program(s) used to solve structure: *SHELXS97* (Sheldrick, 2008 ▶); program(s) used to refine structure: *SHELXL97* (Sheldrick, 2008 ▶); molecular graphics: *SHELXTL* (Sheldrick, 2008 ▶); software used to prepare material for publication: *SHELXL97*.

## Supplementary Material

Crystal structure: contains datablock(s) I, global. DOI: 10.1107/S1600536812043851/lh5547sup1.cif

Structure factors: contains datablock(s) I. DOI: 10.1107/S1600536812043851/lh5547Isup2.hkl

Supplementary material file. DOI: 10.1107/S1600536812043851/lh5547Isup3.cml

Additional supplementary materials:  crystallographic information; 3D view; checkCIF report

## Figures and Tables

**Table 1:** Hydrogen-bond geometry (Å, °)

*D*—H⋯*A*	*D*—H	H⋯*A*	*D*⋯*A*	*D*—H⋯*A*
N—H0*A*⋯O^i^	0.86	2.11	2.921 (2)	157

Symmetry code: (i) 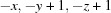.

**Fig. 1.**
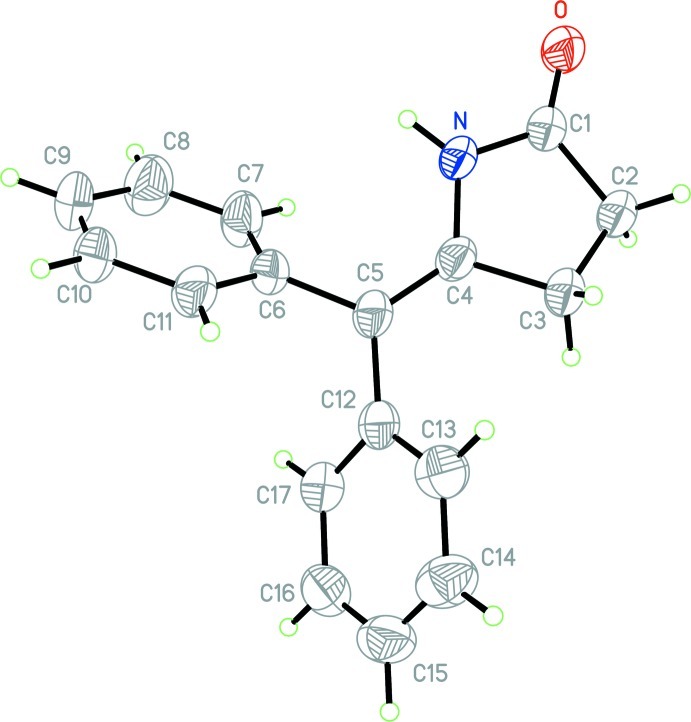
The molecular structure of (I), with ellipsoids for non-H atoms shown at the 50% probability level.

**Fig. 2.**
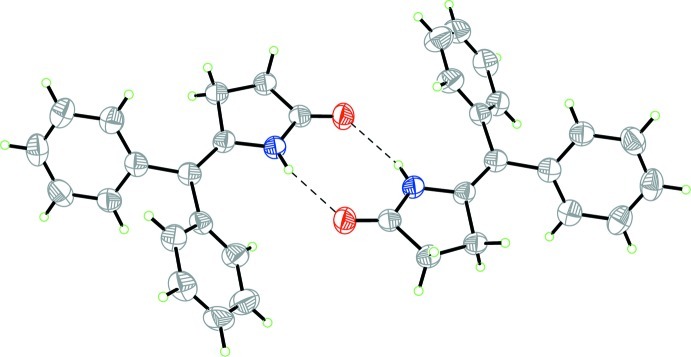
A hydrogen-bonded (dashed lines) dimer of (I).

## supplementary crystallographic information

### Comment

The title compound (I) is the side product obtained from the attempted synthesis of 5-(diphenylmethyl)pyrrolidin-2-one (Fujihara *et al.*, 1999; Enders *et al.*, 2008). We found that adding excess calcium hydride to the reaction mixture could improve the yield of (I). Herein we report the synthesis and crystal structure of the (I). The molecular structure of (I) is shown in Fig. 1. The two phenyl rings form a dihedral angle of 80.1 (2)°. In the crystal, pairs of molecules are linked by N—H···O hydrogen bonds to form inversion dimers (Fig. 2). The intermolecular N—H···O=C hydrogen bonds are similar to those in 3-[(*E*)-benzylidene]indolin-2-one (Asiri *et al.*, 2012).

### Experimental

To a mixture of 5-(hydroxydiphenylmethyl)pyrrolidin-2-one (148 mg, 0.55 mmole) and boron trifluoride etherate (0.4 ml, 3.17 mmole) in 5.5 ml of dichloromethane was added excess calcium hydride. The reaction mixture was stirred at 298 K for 24 h under N_2_ atmosphere. The resulting mixture was partitioned between dichloromethane (10 ml) and H_2_O (10 ml). The organic layer was dried over MgSO_4_ and concentrated *in vacuo*. The residue was separated by chromatography over silica gel and eluted with hexane/ethyl acetate (6/4) to afford 100 mg of the title compound (I) in 73% yield. Single crystals suitable for X-ray measurements were obtained by recrystallization from a dichloromethane/hexane solution of the title compound at room temperature.

### Refinement

All H atoms were placed in geometrically idealized positions and treated as riding on their parent atoms, with C—H = 0.93 or 0.97 Å, N—H = 0.86 Å and *U*_iso_(H) = 1.2*U*_eq_(C, N).

### Crystal data

**Table:**

C_17_H_15_NO	*Z* = 2
*M**_r_* = 249.30	*F*(000) = 264
Triclinic, *P*1	*D*_x_ = 1.254 Mg m^−^^3^
Hall symbol: -P 1	Melting point: 472 K
*a* = 7.135 (2) Å	Mo *K*α radiation, λ = 0.71073 Å
*b* = 7.885 (2) Å	Cell parameters from 1065 reflections
*c* = 12.184 (4) Å	θ = 3.0–29.0°
α = 89.76 (3)°	µ = 0.08 mm^−^^1^
β = 75.09 (3)°	*T* = 293 K
γ = 85.65 (2)°	Parallelpiped, colourless
*V* = 660.4 (4) Å^3^	0.70 × 0.50 × 0.35 mm

### Data collection

**Table:**

Agilent Xcalibur (Sapphire3, Gemini) diffractometer	2999 independent reflections
Radiation source: fine-focus sealed tube	1951 reflections with *I* > 2σ(*I*)
Graphite monochromator	*R*_int_ = 0.052
Detector resolution: 16.0690 pixels mm^-1^	θ_max_ = 29.1°, θ_min_ = 3.0°
ω scans	*h* = −8→9
Absorption correction: multi-scan (*CrysAlis PRO*; Agilent, 2011)	*k* = −10→10
*T*_min_ = 0.542, *T*_max_ = 1.000	*l* = −16→16
5549 measured reflections	

### Refinement

**Table:**

Refinement on *F*^2^	Primary atom site location: structure-invariant direct methods
Least-squares matrix: full	Secondary atom site location: difference Fourier map
*R*[*F*^2^ > 2σ(*F*^2^)] = 0.074	Hydrogen site location: inferred from neighbouring sites
*wR*(*F*^2^) = 0.225	H-atom parameters constrained
*S* = 1.06	*w* = 1/[σ^2^(*F*_o_^2^) + (0.120*P*)^2^] where *P* = (*F*_o_^2^ + 2*F*_c_^2^)/3
2999 reflections	(Δ/σ)_max_ < 0.001
172 parameters	Δρ_max_ = 0.31 e Å^−^^3^
0 restraints	Δρ_min_ = −0.26 e Å^−^^3^

### Special details

**Table:**

Geometry. All e.s.d.'s (except the e.s.d. in the dihedral angle between two l.s. planes) are estimated using the full covariance matrix. The cell e.s.d.'s are taken into account individually in the estimation of e.s.d.'s in distances, angles and torsion angles; correlations between e.s.d.'s in cell parameters are only used when they are defined by crystal symmetry. An approximate (isotropic) treatment of cell e.s.d.'s is used for estimating e.s.d.'s involving l.s. planes.
Refinement. Refinement of *F*^2^ against ALL reflections. The weighted *R*-factor *wR* and goodness of fit *S* are based on *F*^2^, conventional *R*-factors *R* are based on *F*, with *F* set to zero for negative *F*^2^. The threshold expression of *F*^2^ > σ(*F*^2^) is used only for calculating *R*-factors(gt) *etc*. and is not relevant to the choice of reflections for refinement. *R*-factors based on *F*^2^ are statistically about twice as large as those based on *F*, and *R*- factors based on ALL data will be even larger.

### Fractional atomic coordinates and isotropic or equivalent isotropic displacement parameters (Å^2^)

**Table:**

	*x*	*y*	*z*	*U*_iso_*/*U*_eq_	
O	−0.0325 (2)	0.7022 (2)	0.55977 (15)	0.0575 (5)	
N	0.2544 (2)	0.5943 (2)	0.44030 (15)	0.0405 (5)	
H0A	0.2224	0.4921	0.4353	0.049*	
C1	0.1353 (3)	0.7169 (3)	0.50609 (19)	0.0420 (5)	
C2	0.2440 (3)	0.8758 (3)	0.4967 (2)	0.0473 (6)	
H2A	0.2382	0.9216	0.5714	0.057*	
H2B	0.1898	0.9622	0.4543	0.057*	
C3	0.4522 (3)	0.8183 (3)	0.4343 (2)	0.0451 (6)	
H3A	0.5058	0.8992	0.3766	0.054*	
H3B	0.5342	0.8050	0.4865	0.054*	
C4	0.4348 (3)	0.6490 (3)	0.38089 (18)	0.0375 (5)	
C5	0.5618 (3)	0.5669 (3)	0.29326 (18)	0.0384 (5)	
C6	0.5291 (3)	0.3990 (3)	0.24831 (18)	0.0389 (5)	
C7	0.3610 (3)	0.3647 (3)	0.2184 (2)	0.0505 (6)	
H7A	0.2597	0.4492	0.2276	0.061*	
C8	0.3409 (4)	0.2072 (4)	0.1751 (3)	0.0627 (7)	
H8A	0.2263	0.1873	0.1560	0.075*	
C9	0.4876 (4)	0.0803 (3)	0.1600 (2)	0.0590 (7)	
H9A	0.4736	−0.0256	0.1310	0.071*	
C10	0.6556 (4)	0.1125 (3)	0.1885 (2)	0.0543 (7)	
H10A	0.7564	0.0274	0.1785	0.065*	
C11	0.6774 (3)	0.2689 (3)	0.2319 (2)	0.0455 (6)	
H11A	0.7927	0.2878	0.2504	0.055*	
C12	0.7457 (3)	0.6416 (3)	0.23349 (19)	0.0398 (5)	
C13	0.8740 (3)	0.7062 (3)	0.2886 (2)	0.0477 (6)	
H13A	0.8460	0.7022	0.3675	0.057*	
C14	1.0422 (3)	0.7762 (3)	0.2293 (2)	0.0554 (7)	
H14A	1.1248	0.8193	0.2684	0.066*	
C15	1.0873 (3)	0.7819 (3)	0.1129 (2)	0.0581 (7)	
H15A	1.1997	0.8295	0.0728	0.070*	
C16	0.9655 (4)	0.7170 (3)	0.0561 (2)	0.0577 (7)	
H16A	0.9962	0.7195	−0.0228	0.069*	
C17	0.7963 (3)	0.6473 (3)	0.1157 (2)	0.0499 (6)	
H17A	0.7153	0.6036	0.0758	0.060*	

### Atomic displacement parameters (Å^2^)

**Table:**

	*U*^11^	*U*^22^	*U*^33^	*U*^12^	*U*^13^	*U*^23^
O	0.0476 (10)	0.0429 (10)	0.0693 (12)	−0.0101 (7)	0.0101 (8)	−0.0171 (8)
N	0.0425 (10)	0.0255 (9)	0.0486 (11)	−0.0054 (7)	−0.0022 (8)	−0.0062 (8)
C1	0.0452 (13)	0.0324 (12)	0.0446 (12)	−0.0053 (9)	−0.0039 (10)	−0.0067 (9)
C2	0.0496 (14)	0.0324 (12)	0.0553 (14)	−0.0083 (10)	−0.0036 (10)	−0.0102 (10)
C3	0.0464 (13)	0.0327 (12)	0.0519 (14)	−0.0048 (9)	−0.0045 (10)	−0.0102 (10)
C4	0.0385 (11)	0.0258 (11)	0.0471 (12)	−0.0032 (8)	−0.0089 (9)	−0.0018 (9)
C5	0.0414 (12)	0.0249 (11)	0.0464 (12)	0.0024 (8)	−0.0084 (9)	−0.0025 (9)
C6	0.0439 (12)	0.0270 (11)	0.0409 (11)	0.0011 (8)	−0.0030 (9)	−0.0027 (9)
C7	0.0465 (14)	0.0396 (14)	0.0648 (16)	0.0055 (10)	−0.0153 (11)	−0.0127 (11)
C8	0.0571 (16)	0.0543 (17)	0.0803 (19)	−0.0059 (12)	−0.0232 (14)	−0.0149 (14)
C9	0.0744 (18)	0.0344 (14)	0.0628 (17)	−0.0023 (12)	−0.0083 (13)	−0.0152 (11)
C10	0.0607 (16)	0.0270 (12)	0.0652 (16)	0.0085 (10)	−0.0016 (12)	−0.0015 (11)
C11	0.0450 (13)	0.0302 (12)	0.0581 (14)	0.0008 (9)	−0.0088 (10)	−0.0010 (10)
C12	0.0401 (12)	0.0241 (11)	0.0509 (13)	0.0065 (8)	−0.0062 (9)	−0.0026 (9)
C13	0.0438 (13)	0.0441 (14)	0.0544 (14)	0.0011 (10)	−0.0124 (10)	0.0026 (11)
C14	0.0420 (14)	0.0516 (16)	0.0744 (19)	−0.0060 (11)	−0.0176 (12)	0.0064 (13)
C15	0.0418 (13)	0.0478 (15)	0.0752 (19)	−0.0010 (11)	0.0012 (12)	0.0058 (13)
C16	0.0631 (16)	0.0500 (16)	0.0502 (15)	−0.0026 (12)	0.0026 (12)	−0.0028 (12)
C17	0.0515 (14)	0.0410 (14)	0.0532 (14)	−0.0054 (10)	−0.0055 (11)	−0.0088 (11)

### Geometric parameters (Å, º)

**Table:**

O—C1	1.222 (3)	C8—C9	1.369 (4)
N—C1	1.353 (3)	C8—H8A	0.9300
N—C4	1.404 (3)	C9—C10	1.373 (3)
N—H0A	0.8600	C9—H9A	0.9300
C1—C2	1.512 (3)	C10—C11	1.379 (3)
C2—C3	1.520 (3)	C10—H10A	0.9300
C2—H2A	0.9700	C11—H11A	0.9300
C2—H2B	0.9700	C12—C17	1.389 (3)
C3—C4	1.515 (3)	C12—C13	1.393 (3)
C3—H3A	0.9700	C13—C14	1.385 (3)
C3—H3B	0.9700	C13—H13A	0.9300
C4—C5	1.341 (3)	C14—C15	1.373 (4)
C5—C12	1.490 (3)	C14—H14A	0.9300
C5—C6	1.492 (3)	C15—C16	1.369 (4)
C6—C7	1.386 (3)	C15—H15A	0.9300
C6—C11	1.393 (3)	C16—C17	1.390 (3)
C7—C8	1.383 (4)	C16—H16A	0.9300
C7—H7A	0.9300	C17—H17A	0.9300
			
C1—N—C4	113.85 (18)	C9—C8—C7	120.8 (2)
C1—N—H0A	123.1	C9—C8—H8A	119.6
C4—N—H0A	123.1	C7—C8—H8A	119.6
O—C1—N	125.5 (2)	C8—C9—C10	118.6 (2)
O—C1—C2	126.6 (2)	C8—C9—H9A	120.7
N—C1—C2	107.83 (19)	C10—C9—H9A	120.7
C1—C2—C3	104.76 (19)	C9—C10—C11	121.1 (2)
C1—C2—H2A	110.8	C9—C10—H10A	119.4
C3—C2—H2A	110.8	C11—C10—H10A	119.4
C1—C2—H2B	110.8	C10—C11—C6	120.9 (2)
C3—C2—H2B	110.8	C10—C11—H11A	119.5
H2A—C2—H2B	108.9	C6—C11—H11A	119.5
C4—C3—C2	103.89 (16)	C17—C12—C13	116.8 (2)
C4—C3—H3A	111.0	C17—C12—C5	119.22 (19)
C2—C3—H3A	111.0	C13—C12—C5	123.9 (2)
C4—C3—H3B	111.0	C14—C13—C12	121.8 (2)
C2—C3—H3B	111.0	C14—C13—H13A	119.1
H3A—C3—H3B	109.0	C12—C13—H13A	119.1
C5—C4—N	125.92 (19)	C15—C14—C13	120.1 (2)
C5—C4—C3	128.01 (19)	C15—C14—H14A	120.0
N—C4—C3	106.06 (18)	C13—C14—H14A	120.0
C4—C5—C12	121.04 (19)	C16—C15—C14	119.5 (2)
C4—C5—C6	123.16 (19)	C16—C15—H15A	120.2
C12—C5—C6	115.79 (19)	C14—C15—H15A	120.2
C7—C6—C11	117.2 (2)	C15—C16—C17	120.5 (2)
C7—C6—C5	124.03 (19)	C15—C16—H16A	119.8
C11—C6—C5	118.74 (18)	C17—C16—H16A	119.8
C8—C7—C6	121.3 (2)	C12—C17—C16	121.3 (2)
C8—C7—H7A	119.4	C12—C17—H17A	119.3
C6—C7—H7A	119.4	C16—C17—H17A	119.3

### Hydrogen-bond geometry (Å, º)

**Table:**

*D*—H···*A*	*D*—H	H···*A*	*D*···*A*	*D*—H···*A*
N—H0*A*···O^i^	0.86	2.11	2.921 (2)	157

Symmetry code: (i) −*x*, −*y*+1, −*z*+1.

## References

[bb1] Agilent (2011). *CrysAlis PRO* Agilent Technologies, Yarnton, England.

[bb2] Asiri, A. M., Zayed, M. E. M., Ng, S. W. & Tiekink, E. R. T. (2012). *Acta Cryst.* E**68**, o2020. 10.1107/S1600536812024762 PMC3393289 22807846

[bb3] Enders, D. & Han, J. (2008). *Tetrahedron Asymmetry*, **19**, 1367–1371.

[bb4] Fujihara, H. & Tomioka, K. (1999). *J. Chem. Soc. Perkin Trans. 1*, pp. 2377–2382.

[bb5] Sheldrick, G. M. (2008). *Acta Cryst.* A**64**, 112–122. 10.1107/S0108767307043930 18156677

